# Innovation in Newcastle Disease Virus Vectored Avian Influenza Vaccines

**DOI:** 10.3390/v11030300

**Published:** 2019-03-26

**Authors:** Shin-Hee Kim, Siba K. Samal

**Affiliations:** VA-MD College of Veterinary Medicine, University of Maryland, College Park, MD 20742, USA; ssamal@umd.edu

**Keywords:** highly pathogenic avian influenza virus, Newcastle disease virus, live attenuated NDV-vectored vaccines, chimeric NDV vector, poultry

## Abstract

Highly pathogenic avian influenza (HPAI) and Newcastle disease are economically important avian diseases worldwide. Effective vaccination is critical to control these diseases in poultry. Live attenuated Newcastle disease virus (NDV) vectored vaccines have been developed for bivalent vaccination against HPAI viruses and NDV. These vaccines have been generated by inserting the hemagglutinin (HA) gene of avian influenza virus into NDV genomes. In laboratory settings, several experimental NDV-vectored vaccines have protected specific pathogen-free chickens from mortality, clinical signs, and virus shedding against H5 and H7 HPAI viruses and NDV challenges. NDV-vectored H5 vaccines have been licensed for poultry vaccination in China and Mexico. Recently, an antigenically chimeric NDV vector has been generated to overcome pre-existing immunity to NDV in poultry and to provide early protection of poultry in the field. Prime immunization of one-day-old poults with a chimeric NDV vector followed by boosting with a conventional NDV vector has shown to protect broiler chickens against H5 HPAI viruses and a highly virulent NDV. This novel vaccination approach can provide efficient control of HPAI viruses in the field and facilitate poultry vaccination.

## 1. Avian Influenza Vaccines

Avian influenza virus (AIV) is an economically important pathogen of poultry worldwide. The natural reservoirs of the virus are a variety of wild aquatic birds, including ducks, gulls, and shorebirds. The virus can spread from a wide range of aquatic birds to domestic birds causing outbreaks [1,2]. AIV belongs to the family *Orthomyxoviridae* and the genus *Influenzavirus* A. The virus has a negative-sense, single-stranded and segmented RNA genome and contains eight gene segments encoding at least 10 proteins: polymerase basic 1 (PB1), polymerase basic 1 (PB2), polymerase acid (PA), hemagglutinin (HA), nucleoprotein (NP), neuraminidase (NA), matrix 1 (M1), matrix 2 (M2), nonstructural 1 (NS1), and nonstructural 2 (NS2) [3]. The HA and NA proteins are surface glycoproteins and are important for virus infectivity. The HA protein is responsible for virus attachment to the host cell and is the major target of the humoral immune response. The HA protein is synthesized as a precursor protein (HA0) in infected cells and requires cleavage by host cell proteases to transit into an active form (HA1 and HA2). The NA protein plays a role in release and spread of progeny virions by removing sialic acids that are bound by the HA protein. Antibodies against HA inhibit the attachment to the sialic-acid-containing host cell receptor and inhibit fusion between viral and host cell membranes [4]. Antibodies to the NA protein can impede its receptor-destroying function, thus reducing virus replication by inhibiting virus release from infected cells. On the basis of antigenic and phylogenetic differences, 16 HA types and 9 NA types have been identified in viruses isolated from wild waterfowl [5].

Most influenza viruses typically cause asymptomatic infection and little pathology in birds. These low pathogenic avian influenza (LPAI) viruses contain a HA cleavage site that can only be cleaved by proteases available in intestinal and respiratory tracts [6,7]. In contrast, highly pathogenic avian influenza (HPAI) viruses contain multiple basic amino acids at the HA0 cleavage site, resulting in cleavability of the HA protein by ubiquitous intracellular proteases. Therefore, HPAI viruses cause systemic infection and high mortality in chickens and other terrestrial poultry. Two subtypes (H5 and H7) of LPAI viruses can naturally change to a highly pathogenic phenotype through mechanisms during their circulation in domestic poultry, such as acquisition of basic amino acids in the cleavage region of the hemagglutinin (HA) protein by insertion or substitution and recombination with another gene segment(s) or host genome [8,9].

The first line of defense against AI in poultry is focused on biosecurity, education, surveillance, rapid diagnosis, and depopulation of affected flocks to prevent spread of the virus to uninfected flocks [10,11,12]. In addition to good biosecurity, successful vaccination greatly reduces morbidity and mortality of exposed birds and prevents economic losses. The outbreaks involving H5 and H7 subtypes of HPAI have resulted in high mortality in poultry, affecting poultry production and trade [13]. Despite all control efforts, AIV continues to cause outbreaks in poultry and sporadic infection in humans. In the past 20 years, the number of HPAI outbreaks has increased, and the Asian-origin goose/Guangdong (Gs/GD) lineage of H5N1 and Mexican H7N3 lineages of viruses have become endemic in poultry [1]. In addition, the cumulated number of confirmed human cases of H5N1 and Asian H7N9 infection reported to WHO to date is 860 with 454 fatal cases (53% mortality) and 1625 with 623 deaths, respectively [14,15]. Therefore, vaccine development has become a critical component not only for controlling AIV infection in poultry efficiently, but also for preventing the transmission of these viruses from birds to humans. HPAI vaccines have been widely used in China, Egypt, Indonesia, and Vietnam, where H5N1 HPAI is enzootic. Vaccination can also be used as a preventive tool when risk of introduction is high or as an emergency action after an outbreak begins [10,11].

Currently available AI vaccines are not satisfactory. Inactivated AIV vaccines are mostly used for poultry (95% usage) [16]. However, the currently available inactivated vaccines require labor-intensive administration methods and provide suboptimal protection of vaccinated birds [10]. Furthermore, inactivated vaccines can be effective generally when the birds are immunologically mature (>3-week-old ages) [17]. The use of live attenuated AIV vaccines in poultry is not recommended by the World Organization for Animal Health (OIE) due to the potential risk of reassortment or mutations for generating HPAI viruses [13]. Alternatively, replicating viral vector vaccines offer a live vaccine approach without requiring involvement of the complete pathogen or cultivation of the pathogen [18]. Viral vectors offer a number of advantages over traditional vaccines. Viral vector vaccines not only induce outstanding antibody responses, but also elicit cytotoxic lymphocyte (CTL), which is important for control of viral infections. Several viral species have been evaluated as recombinant vectors for AIV vaccines, including Newcastle disease virus (NDV), turkey herpesvirus [19], fowlpox virus [20], adenovirus [21], infectious laryngotracheitis (ILT) virus [22], and Marek’s disease virus (MDV) [23]. The rationale for selecting one vector over another depends upon the quantity of antigen expressed by the vector and the type of cells that produce the antigen following infection by the viral vector. Another factor that influences the choice of vector is the presence of pre-existing immunity to the vector. The use of NDV as a vaccine vector for AIV has multiple advantages over other viral vaccines [24]. The first NDV-vectored vaccine was generated by expressing the major protective antigen, HA protein of human influenza A/WSN/33 (H1N1) [25]. For evaluation of this vaccine candidate for human vaccination against HPAI virus, the immunogenicity of NDV expressing the HA of H5N1 was evaluated in African green monkeys by the intranasal route of administration [26,27]. The NDV-vectored vaccines were highly restricted for replication in the respiratory tract and provided protection against challenge with HPAI viruses, indicating their potential use for human vaccination. This approach has been further evaluated for poultry vaccination against HPAI viruses. Therefore, in this review, we will address the advantages and disadvantages of using NDV vectors for HPAI vaccines, the protective efficacy of H5 and H7 vaccine candidates, and the assessment of a novel NDV vector for early vaccination of poultry in the field.

## 2. NDV Vectors for Live Attenuated AI Vaccines

NDV is a member of the family *Paramyxoviridae* and has a nonsegmented, negative-sense RNA genome that contains six genes (3′-N-P-M-F-HN-L-5′) [28]. NDV strains vary widely in virulence. Based on the severity of the disease in chickens, NDV strains are classified into three pathotypes: lentogenic strains, which cause mild or asymptomatic infections that are restricted to the respiratory tract; mesogenic strains, which are of intermediate virulence; and velogenic strains, which cause systemic infections with high mortality [29]. Naturally occurring lentogenic NDV strains, such as LaSota and B1, are widely used as live attenuated vaccines to control Newcastle disease in poultry with good track records of safety and efficacy. Therefore, a NDV vector carrying the protective antigen of another avian pathogen can be used as a safe bivalent vaccine [28], making it economical and effective for the poultry farmers.

The advent of a reverse genetics system to manipulate the genome of NDV has allowed evaluation of NDV as a vaccine vector against diseases of humans and animals [24]. NDV has a modular genome that facilitates genetic manipulation. This makes it possible to generate vaccine viruses entirely from cloned cDNA [28]. In general, a gene of protective antigen flanked by NDV gene-start and gene-end sequences is inserted into an intergenic noncoding region of an NDV genome as an additional transcription unit. Due to a polar gradient transcription, foreign genes are expressed more efficiently when placed closer to the 3ˊ end of the genome. Although a foreign gene can be placed between any two genes of NDV, the insertion site between the P and M genes has been found to be optimal for efficient expression of the foreign protein and replication of NDV [30,31,32,33]. The insertion of a foreign gene into an NDV genome increases its genome length and gene number, resulting in a growth retardation effect on virus replication and attenuation [34]. NDV accommodates foreign genes (at least 4.5 kb in length) with a good degree of stability.

NDV is an ideal vaccine vector for prevention of HPAI virus infections in poultry. NDV infects via the intranasal route using the same route of infection with AIV. Thus, NDV vector induces both local and systemic immune responses at the respiratory tract [26,27]. The presence of neutralizing antibodies specific for the HA protein at systemic or mucosal sites of infection provides immediate protection against AIV. NDV grows to high titers in cell cultures (10^8^ pfu/ml) and in embryonated eggs (10^9^ pfu/ml), thus making it cost-effective [28]. The vaccine can potentially be administered using automated methods as a spray or in drinking water, since Newcastle disease vaccines have often been administered by using these approaches. Further, NDV vector is feasible to update protective antigens with replaceable vaccine cassettes of currently circulating virus in the field. Therefore, it provides a convenient platform for field AIV vaccination.

## 3. NDV-Vectored Vaccines against H5 HPAI Viruses

Gs/GD lineage of H5N1 HPAI has spread to over 70 countries since 1997 and is currently endemic in poultry in at least eight different countries [35,36]. H5 HPAI viruses have also caused sporadic zoonotic infections. The viruses have become genetically diversified represented by multiple phylogenetic lineages, classified as clade 0 to 9 [37]. Currently, viruses from clades 0, 1, and 2 have infected humans since the initial 1997 outbreaks [8,38,39]. NDV has been used to express the HA proteins from clades 1 and 2 of H5 HPAI viruses (Table 1). Heterologous challenge could provide a partial protection for vaccinated chickens [40]. Therefore, NDV-vectored vaccines also need to be updated with the HA protein from emerging H5 HPAI viruses. The protective efficacy of vaccines has been dependent on the expression levels of the HA protein, the location of the HA gene inserts in the NDV genome, and the ages of the immunized chickens. The HA gene has been inserted between the P and M and between the M and F genes in NDV genome. Most studies agree that the insertion site between the P and M is the optimal location for high levels of HA protein expression. To address a safety concern, NDV-vectored vaccines were further generated by replacing the polybasic cleavage site in HPAIV HA with that from a low-pathogenicity strain of influenza virus [26].

The protective efficacy of NDV-vectored H5 vaccines have been mostly evaluated in specific pathogen-free (SPF) chickens. A single immunization of chickens at the ages of two weeks or older has resulted in the protection of chickens from mortality after the HPAI virus challenge [41,42,43]. The levels of virus shedding were variable depending on the vaccine construct and dose of the challenge viruses. In general, most studies showed protection of chickens from shedding of challenge viruses. A few studies also evaluated vaccine efficacy by immunizing one-day-old chicks. The mortality rates of challenged chickens were dependent on the immunization doses [44]. All challenge control birds died at 2 dpi. The vaccination of chickens with 10^5^ EID_50_ and 10^6^ EID_50_ resulted in 100% and 10% of mortality at 5 dpi, respectively.

Avirulent NDV strain LaSota has been mostly used for vaccine development (Table 1). Velogenic NDV strain has also been used by attenuating the virus. Specifically, the polybasic cleavage site of the fusion (F) protein of velogenic NDV strain Herts/33 was modified into a monobasic cleavage motif (GRQGR↓L) [45]. The attenuated virus was subsequently used as a vector for vaccinating chickens against HPAI virus. Immunization of chickens with this NDV vaccine provided 100% (intramuscular route) and 80% (ocularnasal/ intratracheal route) of protection against a lethal challenge with H5N1 HPAI. As shown in this study, intramuscular immunization can induce better protective immunity than the oculanasal route of immunization. Therefore, to enhance the protective immunity, intramuscular immunization has been alternatively used for H5 clade 2.3.4.4 HPAI challenge study [46]. After double doses of immunization, NDV-H5 vaccines protected chickens from lethal challenge with the highly pathogenic H5N2 A/turkey/Minnesota/9845-4/2015 virus. Only minimal virus shedding was observed in vaccinated groups.

## 4. NDV-Vectored Vaccines against H7 HPAI Viruses

In general, H7 subtype viruses have low immunogenicity [49]. The NDV vector has also shown to induce relatively low levels of H7 HA-specific antibodies in SPF chickens [50]. Various strategies have been used to enhance the protective immunity, such as modified NDV vectors, intramuscular routes of immunization, and booster immunization. In addition, the ectodomain of HA protein from A/chicken/NY/13142-5/94 H7N2 LPAI virus was fused with the transmembrane and cytoplasmic domains derived from the F protein of NDV [51]. This approach resulted in the enhanced incorporation of the foreign protein into virus particles and the protection of chickens against both HPAI virus and highly virulent NDV.

NDV strain Hitchner B1 was the first NDV vector used to generate avian influenza vaccine by expressing the H7N2 HA protein (rNDV-AIV-H7) [50]. Groups of two-week-old SPF white Leghorn chickens were vaccinated with rNDV-AIV-H7 and challenged two weeks later with viscerotropic velogenic NDV or HPAI virus. The vaccine provided partial protection (40%) from NDV and HPAI virus challenges. To enhance the protective efficacy, the fusogenic rNDV strain B1 vector was generated by replacing the cleavage site of the F protein with one containing two extra arginine residues [51]. The ectodomain of the HA protein was expressed by this fusogenic rNDV vector. A single immunization of chickens showed enhanced protective efficacy with a 90% protection against a heterologous H7N7 HPAI virus and a complete protection against a highly virulent NDV. However, the modified B1 vector with multiple basic residues at the F protein cleavage site is considered to be a Select Agent, and therefore cannot be used as a vaccine vector.

Since 2013, the emergence of novel H7N9 viruses have been a threat to public health by causing severe human infections with high mortality in China, although the viruses have only low pathogenicity in chickens [52]. Therefore, an NDV-vectored vaccine was generated by expressing the HA protein of a human isolate of H7N9 (Anhui/1/2013) [48]. NDV-H7 induced low levels of hemagglutination inhibition (HI) antibodies after the first vaccination via the intramuscular route or oculanasal route, and boost immunization was required to enhance HI antibody titers (1:40 to 1:160). The challenge with Anhui/1/2013 H7N9 did not induce clinical signs and mortality in both mock-vaccinated and vaccinated chickens due to the low pathogenicity of H7N9 virus in chickens. Virus shedding was detected in more than half of the mock-vaccinated chickens via the oropharynx at 1 and 3 dpi. In contrast, virus shedding was efficiently controlled in the NDV-H7-intramuscularly or -oculonasally vaccinated chickens.

In 2017, H7N9 HPAI viruses emerged resulting from an insertion of four basic amino acids in the HA proteolytic cleavage site [53]. The outbreaks of HPAI H7N9 virus in poultry in China have resulted in the depopulation of over 800,000 chickens. A NDV-vectored vaccine was generated by using a genetically attenuated currently circulating genotype VII NDV strain [54]. The protective antigen was the HA protein from an LPAI H7N9 strain. The NDV-vectored vaccine by the intranasal route of immunization elicited undetectable levels of HI and virus neutralization titers but high IgY antibody titers against H7N9 measured by ELISA. The vaccine provided 80% protection against HPAI H7N9 challenge with reduced viral shedding and a complete protection from a highly virulent NDV challenge.

Recently, the number of H7 HPAI outbreaks has increased in a wide geographic location. Efforts have been made to generate live attenuated NDV-vectored vaccines. In contrast to H5 vaccines, most NDV-vectored H7 vaccines were unable to provide 100% of protection from mortality, probably due to low immunogenicity of the H7 HA protein. This suggests that NDV-vectored H7 vaccines require custom tailored approaches with a proper selection of protective antigens, vectors, and vaccination schemes for effective vaccination.

## 5. Use of NDV-Vectored Avian Influenza Vaccines in the Field

The suboptimal protection of poultry due to poor quality of inactivated vaccines and improper vaccination has resulted in continuous circulation of AIVs in the field [16]. The administration of prime-and-boost vaccination is especially challenging with numerous birds in industrial settings and rapid turnover rates of poultry populations [10]. A high labor cost for administration is also a disadvantage of inactivated vaccination in developed countries. Although vaccination of broiler poultry is necessary, a long-withdrawal period for inactivated vaccines has limited AIV vaccination in hatcheries.

To facilitate the administration of AIV vaccines, live-attenuated vectored vaccines have been licensed for poultry use. Recombinant NDVs are approved as bivalent vaccines in poultry against NDV and AIV, and are used in China for H5N1 HPAI viruses [56] and in Mexico for the H5N2 LPAI viruses [57]. In China, the NDV vaccines contained influenza virus HA sequences from GS/GD/96 (rLH5-1), A/bar-headed goose/Qinghai/3/05 (rLH5-3), and CK/SX/06 virus (rLH5- 4). These NDV-vectored vaccines induced HI antibody responses to NDV and to H5 avian influenza viruses in chickens and protected chickens from morbidity, mortality, and virus shedding after challenges with HPAI viruses and NDV [58]. In 2006, rLH5-1 was approved for chicken vaccination. In 2008, the vaccine was updated to rLH5-5 expressing the HA protein from A/duck/Anhui/1/06 (clade 2.3).

NDV is also an important avian pathogen worldwide [28]. Almost all commercial chickens and turkeys are vaccinated against NDV, and poults typically have high levels of maternal antibody, which could interfere with the protective efficacy of the NDV-vectored vaccine in the field [59,60]. This obstacle was also observed with other vector viruses, such as fowlpox virus [61] and turkey herpesvirus [62,63]. Therefore, NDV-vectored vaccines may not be highly effective in commercial chickens less than two-weeks-old because maternal antibodies to NDV can restrict their replication [64]. This is a significant drawback because chickens are susceptible to HPAI virus infection in the first four weeks of life. Currently, the use of NDV-vectored AIV vaccines is limited in the field.

## 6. A New Platform for NDV Vectors to Overcome Pre-Existing Vector Immunity in Poultry

**Figure 1:**
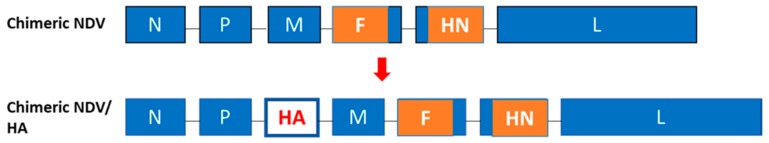
Generation of chimeric NDV-vectored vaccines. Ectodomains of the F and HN genes derived from APMV-2 are shown as orange rectangles. The HA gene was placed between the P and M of the chimeric NDV genome.

The protective efficacy of NDV-vectored vaccines has been mostly evaluated by immunizing SPF chickens (Table 1 and Table 2). These experimental studies cannot completely simulate field conditions [10,60]. Consequently, there has been a discrepancy in protective efficacy of vaccine trials between the laboratory and field conditions. To overcome maternal immunity to NDV, an antigenically distinctive chimeric NDV vaccine vector was generated by replacing the ectodomains of NDV F and HN proteins with those of avirulent avian paramyxovirus serotype-2 (APMV-2) (Figure 1) [65]. NDV and APMV-2 represent two distinct serotypes, and their F and HN glycoproteins have 41% and 35% amino acid sequence identity, respectively. The vector has the antigenicity of APMV-2. The chimeric vector was constructed due to inefficient replication of APMV-2 and incapable of accommodating a large size of foreign gene.

The chimeric vector was comparable to the conventional NDV vector by stably accommodating the HA gene in the genome and expressing the protein at high levels when placed between the P and M genes. The immunization of chickens with chimeric NDV resulted in 100% mortality of chickens after a challenge with a highly virulent NDV strain, thus demonstrating that the chimeric NDV is antigenically distant from NDV. This suggested that a maternal antibody to NDV could not inhibit the replication of chimeric vaccine virus. The chimeric vaccine virus was highly attenuated compared to LaSota vectored vaccine, since the F and HN proteins play a major role in the viral pathogenesis [66,67]. The chimeric NDV expressing the HA protein from A/Vietnam/1203/2004 H5N1 (clade 1) provided a partial protection of one-day-old immunized chickens against homologous H5N1 challenge, indicating its potential use for early protection of chickens.

## 7. Use of Chimeric NDV Vectors for a Heterologous NDV-Vectored Vaccination in Poultry

The protective efficacy of chimeric NDV expressing the HA protein was enhanced by generating three chimeric NDV-vectored vaccine candidates expressing the HA protein in combination with the NA protein, matrix 1 (M1) protein, or nonstructural 1 (NS1) protein [68]. Recovery of all three viruses demonstrated that the chimeric NDV vector can accommodate two foreign genes. Despite the increase in genome length, all the vaccine viruses replicated efficiently in vitro and in vivo. Among the candidate vaccines, prime with chimeric NDV/ HA-NA followed by LaSota/ HA boost protected broiler chickens against mortality, clinical signs, and shedding of A/Vietnam/1203/2004 H5N1. In this study, one-day-old chicks were retrieved from a parent flock in a commercial hatchery that had received routine vaccinations, including NDV vaccine. The findings of this study suggest that a heterologous prime-boost vaccination strategy can circumvent pre-existing maternal antibodies to NDV and provide better protection against the HPAI virus. This approach also protected chickens against a highly virulent NDV strain GB Texas (GBT), suggesting potential use as a dual vaccination in the field.

**Figure 2:**
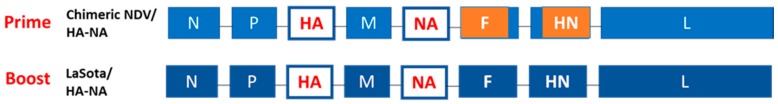
Generation of chimeric NDV- and LaSota-vectored vaccine viruses. The HA and NA genes were placed between the P and M genes and between the M and F genes, respectively. Ectodomains of the F and HN genes derived from APMV-2 are shown as orange rectangles.

H5 HPAI viruses (clade 2.3.4.4) emerged in 2014 and spread by migratory birds into wide geographic regions (Asia, Europe, and North America) [69,70]. The outbreaks of H5 viruses have also caused economic losses in commercial poultry (e.g., an estimated $3.3 billion in economic losses in the U.S.) The heterologous NDV vaccination strategy was further used for evaluation of protective efficacy of H5 clade 2.3.4.4 HPAI vaccines in broiler chickens [71]. Chicks were immunized with chimeric NDV expressing the HA and NA proteins from A/Northern Pintail/WA/40964/2014 H5N2 (prime) and subsequently with LaSota/ HA-NA (boost) (Figure 2). This study found the induction of vector-specific antibody titers by chimeric NDV/ HA-NA vaccine in two-week-old chickens. However, boosting with LaSota vector did not induce high titers of LaSota-specific antibody in four-week-old chickens due to pre-existing NDV vector immunity in broiler chickens. This is mainly due to the persistence of NDV vector immunity in progeny of high titer breeders. However, H5-specific HI serum titers were significantly enhanced in broiler chickens at 2 weeks post-boosting. This could be due to the synergistic effect of lasting immunity with chimeric NDV/ HA-NA vaccine and boost immunity with LaSota/ HA-NA vaccine on protective efficacy. Subsequently, a challenge experiment showed complete protection of broiler chickens from mortality, morbidity, and shedding of HPAI viruses (H5N2 and H5N8) and NDV strain GBT.

A single immunization makes it convenient for vaccination. However, additional boosting would be a practical approach for poultry vaccination in those field conditions where the birds can be immunosuppressed from other infectious agents and/or environmental stress [10,72]. Compared to boosting with inactivated AIV vaccines, a heterologous NDV boost strategy would provide improved immunization with a convenient platform. First, use of antigenically different live vectored vaccines can be cost-effective and potentially applicable for mass vaccination through spray in the hatchery or drinking water. Boosting with LaSota-vectored vaccines can also be used as a dual vaccination purpose. Second, this approach can induce a robust immune response to AIV by a replicating virus used for immunization. Therefore, this vaccination strategy has potential to be used for practical and effective vaccination in the field.

## 8. Conclusions

NDV vectors have several advantages to be commercially used as a bivalent vaccine against HPAI virus and NDV. The live attenuated NDV vaccines have a proven track record of safety and protective efficacy in the field. NDV-vectored H5 and H7 vaccines have already been evaluated in the field. Recently, a heterologous NDV-vectored vaccination approach was shown to be useful for the early vaccination of chickens in the field. This novel vector platform can circumvent the maternal antibody issue and also can be used for other economically important avian pathogens, such as ILT and infectious bronchitis viruses. Therefore, NDV-vectored vaccination can provide a practical strategy for rapid, efficient, and economical immunization of poultry in the field.

## Figures and Tables

**Table 1:** Newcastle disease virus (NDV)-vectored vaccines against H5 serotypes.

Origin of the HA Protein	NDV	Immunized Host (age)	Route of Immunization	Reference
A/chicken/Italy/8/98 H5N2	LaSota	SPF chickens (3 wk)	on, 10^6^ EID_50_	[41]
A/Vietnam/1203/2004 H5N1	LaSota	SPF chickens (2 wk)	on, 10^6^ EID_50_	[42]
A/Vietnam/1203/2004 H5N1	LaSota	SPF chickens (1-day-old)	on, 10^6^ EID_50_	[44]
A/Vietnam/1194/2004	Herts/33	SPF chickens (6 wk)	im, 10^7^ TCID_50_	[45]
A/Bar-headed goose/Qinghai/3/2005 H5N1	LaSota	SPF chickens (1 wk)	on, 10^6^ EID_50_	[47]
A/chicken/Bali/U8661/2009 (clade 2.1.3.2)	LaSota	SPF chickens 6 wk and 8 wk)	on, 5 × 10^6^ PFU/bird	[48]
A/chicken/Iowa/04-20/2015 H5N2 (clade 2.3.4.4)	LaSota	SPF chickens (2 wk and 4 wk)	im, 5 × 10^6^ TCID_50_	[46]

on: oculonasal, im: intramuscular, wk: week, EID_50_: 50% embryo infectious dose, TCID_50_: 50% tissue culture infective dose.

**Table 2:** NDV-vectored vaccines against H7 serotypes.

Origin of the HA Protein	NDV	Immunized Host (age)	Route of Immunization	Reference
A/chicken/NY/13142-5/94 (H7N2)	B1	SPF chickens (2 wk)	Eyedrop, 10^6^ EID_50_	[50]
A/chicken/NY/13142-5/94 (H7N2)	Fusogenic B1	SPF chickens (2 wk and 4 wk)	Eyedrop, 10^6^ EID_50_	[51]
A/chicken/Italy/445/99 (H7N1)	LaSota	SPF chickens (3 wk)	on, 10^6^ EID_50_	[55]
Anhui/1/2013 (H7N9)	LaSota	SPF chickens (6 wk)	im, 5 × 10^6^ PFU	[48]
A/chicken/Zhejiang/JX148/2014 (H7N9)	LX	SPF chickens (3 wk and 5wk)	in, 5 × 10^6^ EID_50_	[54]

on: oculonasal, im: intramuscular, in: intranasal, EID_50_: 50% embryo infectious dose.
